# Practice Patterns in Diagnosis and Treatment of Lower Urinary Tract Symptoms due to Benign Prostatic Hyperplasia among General Practitioners: Lessons Learnt from a Greek Survey

**DOI:** 10.4274/balkanmedj.2017.1659

**Published:** 2018-05-29

**Authors:** Michael Samarinas, Kyriakos Moysidis, Pigi Perdikaki, Stavros Gravas

**Affiliations:** 1 Department of Urology, University of Thessaly School of Medicine, Larissa, Greece; 2 Department of Urology, Aristotle University of Thessaloniki School of Medicine, Thessaloniki, Greece; 3 Chair of the Hellenic College of General Practitioners, Health Center of Astros, Astros, Greece

To the Editor,

Male lower urinary tract symptoms, which are prevalent and directly related to aging process, are a concern and impair the quality of life (1). The role of general practitioners is crucial for the management of male lower urinary tract symptoms to improve healthcare and reduce the associated costs. Recording the practice patterns for the diagnosis and treatment of male lower urinary tract symptoms is not common in the real world (2,3,4). Large differences have been found in the diagnostic workup and in the application of clinical guidelines. We performed a survey study to assess the practice patterns among general practitioners in Greece for the management of male lower urinary tract symptoms associated with benign prostatic hyperplasia and to compare the practice patterns with current guidelines. A total of 1083 general practitioners were invited to fill in a questionnaire on demographic data, diagnostic tests, and therapeutic choices made for male lower urinary tract symptoms. Different clinical scenarios were investigated, and the response rate was 23.3% comprising 135 males and 117 females. The majority (71.4%) of general practitioners were aged below 50 years, and 24.6% of them were practicing in rural areas. The European Association of Urology guidelines recommend medical history, physical examination, symptom questionnaires, urinalysis, post-void residual measurement, and prostate-specific antigen test for the initial evaluation of male lower urinary tract symptoms (1). Medical history and urinalysis are present in the Greek general practitioners’ diagnostic armamentarium for male lower urinary tract symptoms, but surprisingly, only half of them reported the use of digital rectal examination. In addition, only half of them would ask for a prostate-specific antigen test and a lesser proportion (33%) preferred post-void residual measurement (Table 1). These findings indicate a lack of compliance with guidelines. Table 1 also shows the differences with the results from the most recent study in five European countries (2). Interestingly, referral to a urologist for further evaluation and treatment was the first approach after diagnosis (38.1%). Subgroup analysis revealed that general practitioners younger than 50 years of age referred men with lower urinary tract symptoms to urologists for further management more frequently, compared with older general practitioners (p<0.001) (3). General practitioners who were working in urban areas also referred patients with male lower urinary tract symptoms to a urologist more frequently than those from rural areas (p<0.001) (4). Regarding the treatment choices, the most common therapeutic choices were monotherapy with an α-blocker (48.6%) or 5α-reductase inhibitors (30.6%) or their combination (18.9%). The responses to different case scenarios showed that the therapeutic approach did not differ. However, 5α-reductase inhibitors were administered to a significant percentage of patients with small prostates, ranging from 37.9% to 76.2% in different scenarios, although this is contraindicated by the European guidelines (1). The reasons for the observed discrepancy between guidelines and clinical practice include the lack of high-level evidence for the investigational tests; the differences in primary care provision, beliefs, costs, availability, reimbursement policy, and practice of defensive medicine; and the uncertainty about how to proceed with specific patients (5). In conclusion, there is a relative lack of compliance with the guidelines on male lower urinary tract symptoms management among Greek general practitioners, while age and area of practice are the factors that affect the decision to treat or refer patients. These findings underline the urgent need for programs dedicated to general practitioners’ training. Such initiatives will be beneficial for both physicians and patients who require urological investigation and therapy.

## Figures and Tables

**Table 1:**
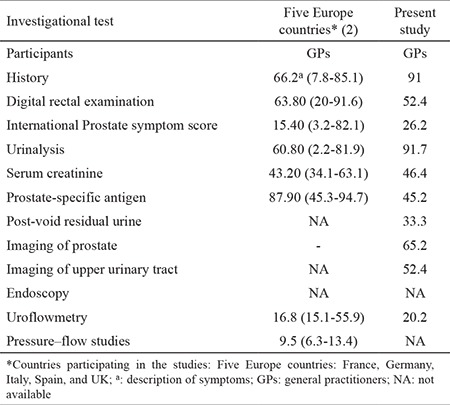
Investigational tests used in a primary setting for the assessment of lower urinary tract symptoms in the present study and in Europe (mean % and range)
